# Physical exercise, autophagy and cardiometabolic stress in aging

**DOI:** 10.18632/aging.102129

**Published:** 2019-07-29

**Authors:** Haili Tian, Peijie Chen, Jun Ren

**Affiliations:** 1 School of Kinesiology, Shanghai University of Sport, Shanghai 200438, China; 2 Center for Cardiovascular Research and Alternative Medicine, University of Wyoming College of Health Sciences, Laramie, WY 82071, USA

**Keywords:** metabolic stress, physical exercise, aging, autophagy, cardiovascular

The average human life expectancy increased significantly over the past decades, resulting in a rapid-growing aging population in our societies [1]. Not surprisingly, approaches to maintain healthy aging and reduce aging-associated cardiovascular morbidity and mortality become a burning issue for global health care. The recent surge in metabolic diseases such as dyslipidemia, obesity, insulin resistance, hypertension and type 2 diabetes mellitus also drastically promoted premature aging and reduced life expectancy [2]. A close tie has been confirmed between obesity and shortened life span or years of life lost [3]. On the other side of the coin, aging serves as a major determinant for metabolic anomalies given the susceptibility of the elderly to various metabolic risk factors [4]. Premature cardiovascular aging develops in patients with metabolic diseases and is predominantly manifested as pathological cardiac remodeling, decreased cardiac pump function, vascular inflammation, stiffness, calcification and endothelial injury [5]. Clinical evidence denoted a high prevalence of cardiovascular events such as myocardial infarction or stroke in patients with progeria (premature aging) syndrome [6]. Up-to-date, several theories have been indicated for cardiovascular aging, such as DNA damage, oxidative stress, telomere shortening, apoptosis, and compromised autonomic response. In addition, other factors may also contribute to the tie between metabolic stress and longevity including behavior, gender, causality versus correlation, and experimental models (primates versus rodents). With the huge economic impact from cardiovascular diseases such as coronary heart disease, arterial hypertension, stroke and heart failure on our society and global health care, better knowledge of the precise molecular mechanisms and treatment remedies are pertinent for premature cardiovascular aging.

**Figure 1:**
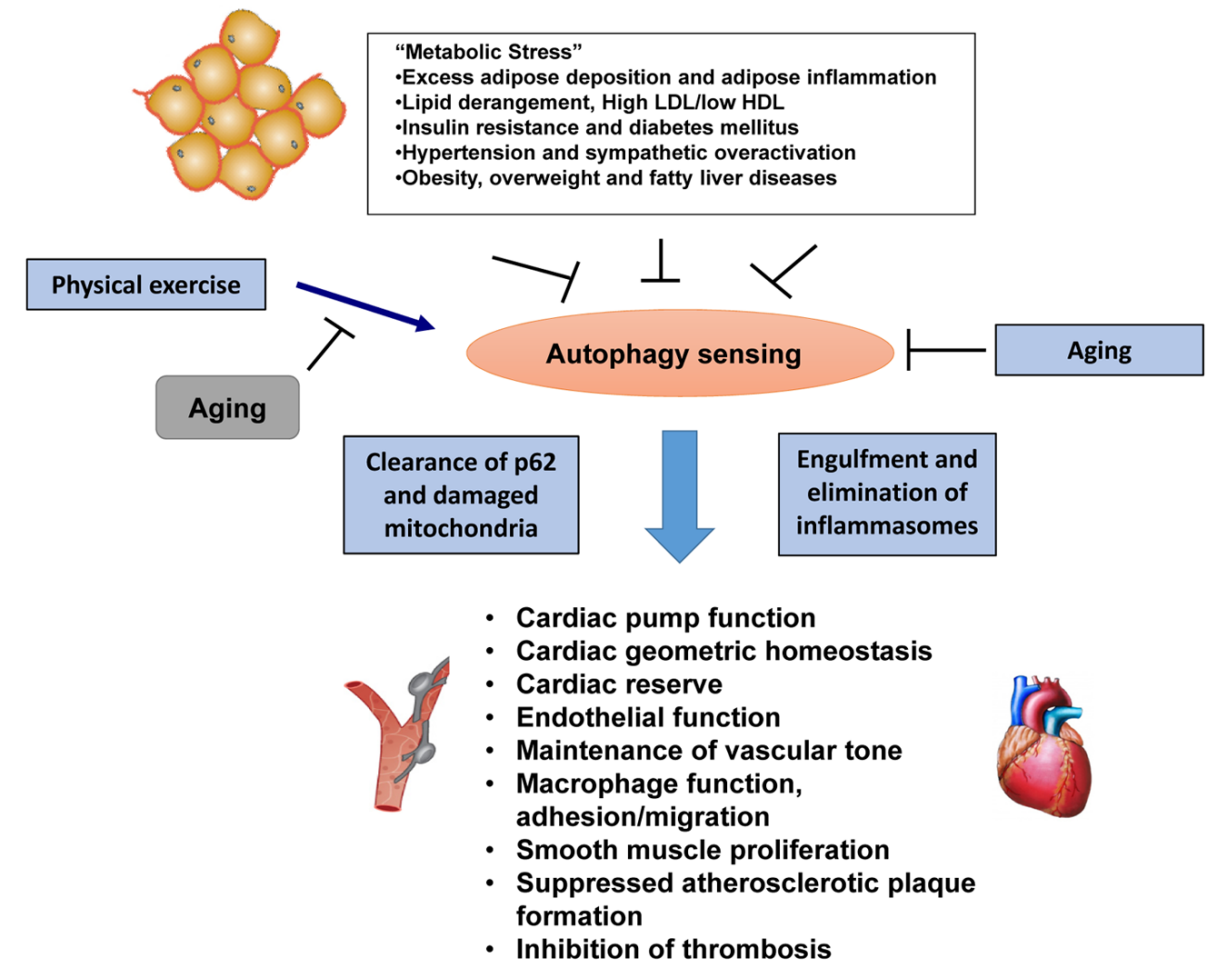
**Metabolic stress and exercise on cardio-vascular function in aging.** Schematic diagram displaying metabolic stress and physical exercise on cardiovascular function, and the potential impact of aging on exercise-elicited beneficial responses.

Although a number of pharmacotherapies were reported for “rejuvenation” and longevity [1], none seems to be able to radically delay or reverse the progression of cardiovascular aging. In addition to pharmacological therapy, lifestyle modifications including weight loss and physical exercise received much attention in correcting metabolic anomalies, improving cardio-vascular health and retarding premature aging. For example, other than the well-known lifespan extension, calorie restriction promotes telomerase activity, improves insulin sensitivity, decreases adiposity, and benefits cardiac pump function. Much of caloric restriction-induced beneficial response on cardio-vascular aging is believed to be mediated through autophagy, a highly conserved cellular process to degrade and recycle the long-lived and damaged cellular organelles, nutrients and debris [1]. Likewise, physical exercise (yet another means to induce autophagy) also improves general health and decreases cardiovascular risks through autophagy induction. However, the benefit of habitual or daily exercise on cardiovascular health is less clear for older populations with mobility limitations that restrict their ability to engage in physical exercise (Figure 1). Prospective studies mainly emphasized on exercise participation, with limited scopes on exercise intervention and reduction of cardiovascular events in the elderly. Using accelerometry as an objective means to assess activity levels, it was shown that physical activity was associated with incidence of cardiovascular events among older adults with limited mobility [7]. These data revealed the risk of experiencing a cardiovascular event possibly based on habitual activity levels, without pinpointing risk reduction and exercise intervention. A more recent prospective training study has provided closer scrutiny to the impact of physical exercise intensity and human aging [8]. These researchers evaluated endurance, interval and resistance trainings on telomerase activity and telomere length and found that endurance training (continuous running) and interval training (4 x4 method), but not resistance training (circle training on 8 devices) increased telomerase activity and telomere length, essential markers for cellular senescence, regenerative capacity and healthy aging. These data favor the notion that not all physical exercise activities would fare the same for aging (although cardiovascular aging was not examined here). Moreover, improved glucose metabolism was noted with regular exercise in older individuals, attributing to favorable body composition including reduced body fat. These findings implicated that older populations should benefit from regular physical activity (but unlikely those with high intensity) to reduce the risk of premature aging and metabolic anomalies. Nonetheless, levels of autophagy were not examined in collaboration with various degrees of physical exercise intensity.

In an era of unprecedented nutrition excess, balanced nutrition helps to maintain lifespan and healthspan. Nonetheless, these nutritional stresses prompt metabolic derangements (e.g., obesity, insulin resistance, inflammation, and circadian rhythm disorder) and disturbs autophagy that would compromise the “healthy” aging process. Ample evidence has denoted an essential role for compromised autophagy in cardiovascular aging [1,4]. Deficiency in autophagy dampens whereas autophagy induction benefits cardiovascular function in aging models through regulation of clearance of misfolded proteins, damaged DNA, and defective mitochondria. Metabolic stress disturbs autophagy to facilitate the transition of “healthy” into a pre-senescent state with overt tissue and cellular pathology. Although physical exercise may offer benefits in autophagy induction and cardiovascular function, caution has to be taken in the aging population where autophagy reserves are already compromised. Future efforts should be engaged on the precise regulation of physical exercise on autophagy regulation to retard or halt aging-associated cardiovascular anomalies. These approaches may provide some useful guidance for exercise intervention on cardiovascular disease in the elder populations.
